# A phase II study of sequential neoadjuvant gemcitabine plus doxorubicin followed by gemcitabine plus cisplatin in patients with operable breast cancer: prediction of response using molecular profiling

**DOI:** 10.1038/sj.bjc.6604322

**Published:** 2008-04-01

**Authors:** P K Julka, R T Chacko, S Nag, R Parshad, A Nair, D S Oh, Z Hu, C B Koppiker, S Nair, R Dawar, N Dhindsa, I D Miller, D Ma, B Lin, B Awasthy, C M Perou

**Affiliations:** 1 Department of Radiotherapy and Oncology, AIIMS, New Delhi 110029, India; 2 Department of Medical Oncology, Christian Medical College, Vellore, Tamil Nadu 632004, India; 3 Department of Medical Oncology, HCJMRI, Pune, Maharashtra 411001, India; 4 Departments of Genetics and Pathology and Laboratory Medicine, Lineberger Comprehensive Cancer Center, University of North Carolina at Chapel Hill, Chapel Hill, NC 27599, USA; 5 Eli Lilly and Company (India) Pvt. Ltd., Gurgaon, Haryana 122001, India; 6 Department of Pathology, Aberdeen Royal Infirmary, Foresterhill, Aberdeen AB25 2ZD, UK; 7 Eli Lilly and Company, Indianapolis, IN 46285, USA; 8 Health Care Global Enterprises, Curie Centre of Oncology, St John's Hospital Campus, Koramangala, Bangalore 560034, India

**Keywords:** breast cancer, chemotherapy, gemcitabine, gene expression, microarrays, neoadjuvant therapy

## Abstract

This study examined the pathological complete response (pCR) rate and safety of sequential gemcitabine-based combinations in breast cancer. We also examined gene expression profiles from tumour biopsies to identify biomarkers predictive of response. Indian women with large or locally advanced breast cancer received 4 cycles of gemcitabine 1200 mg m^−2^ plus doxorubicin 60 mg m^−2^ (Gem+Dox), then 4 cycles of gemcitabine 1000 mg m^−2^ plus cisplatin 70 mg m^−2^ (Gem+Cis), and surgery. Three alternate dosing sequences were used during cycle 1 to examine dynamic changes in molecular profiles. Of 65 women treated, 13 (24.5% of 53 patients with surgery) had a pCR and 22 (33.8%) had a complete clinical response. Patients administered Gem d1, 8 and Dox d2 in cycle 1 (20 of 65) reported more toxicities, with G3/4 neutropenic infection/febrile neutropenia (7 of 20) as the most common cycle-1 event. Four drug-related deaths occurred. In 46 of 65 patients, 10-fold cross validated supervised analyses identified gene expression patterns that predicted with ⩾73% accuracy (1) clinical complete response after eight cycles, (2) overall clinical complete response, and (3) pCR. This regimen shows strong activity. Patients receiving Gem d1, 8 and Dox d2 experienced unacceptable toxicity, whereas patients on other sequences had manageable safety profiles. Gene expression patterns may predict benefit from gemcitabine-containing neoadjuvant therapy.

Although the effectiveness of neoadjuvant chemotherapy *vs* adjuvant chemotherapy in locally advanced breast cancer remains controversial (Estévez and Gradishar, 2004; Cianfrocca and Gradishar, 2005), general findings indicate that both approaches result in equivalent disease-free survival and overall survival (Scholl *et al*, 1994; Fisher *et al*, 1998; van der Hage *et al*, 2001). Clinical trial results indicate that 60–80% of women who receive neoadjuvant chemotherapy have a clinical response, and 10–20% achieve a pathological complete response (pCR) (Cianfrocca and Gradishar, 2005). Because pCR predicts long-term survival (Fisher *et al*, 1998; Kuerer *et al*, 1999; Wolmark *et al*, 2001), ongoing research is focused on identifying regimens that improve the pCR rate and on identifying biomarkers that can predict response to neoadjuvant chemotherapy.

Anthracycline-based regimens are efficacious and widely used in neoadjuvant breast cancer therapy, but debate remains as to which combinations provide encouraging activity balanced by an acceptable safety profile. Gemcitabine has demonstrated single-agent activity in breast cancer (Heinemann, 2003), and is indicated as first-line therapy in combination with paclitaxel for patients with metastatic breast cancer after failure of prior anthracycline-containing adjuvant chemotherapy (Albain *et al*, 2004). In the neoadjuvant setting, gemcitabine has been combined with anthracyclines and/or taxanes in several phase II studies of locally advanced breast cancer, with clinical response rates and pCR rates ranging from 71–95% and 3–26%, respectively (Gomez *et al*, 2001; Silva *et al*, 2002; Estévez *et al*, 2004; Schneeweiss *et al*, 2004).

Gemcitabine plus cisplatin (Gem+Cis) combination therapy has also shown activity in metastatic or refractory breast cancer, with response rates from 26–50% (Heinemann, 2002). Given the tumour shrinking capability of the combination of gemcitabine and doxorubicin (Gem+Dox) (Pérez-Manga *et al*, 2000; Gomez *et al*, 2001), we hypothesised that additional cycles of combination chemotherapy may improve pCR rates. In this phase II study, we evaluated two combination regimens administered sequentially as neoadjuvant chemotherapy in Indian women with operable breast cancer: four cycles of Gem+Dox followed by four cycles of Gem+Cis.

Administering chemotherapy prior to surgical resection provides an opportunity for translational research. By collecting pretreatment biopsies, we attempted to identify biomarkers that could predict chemotherapy response, whereas collecting post-treatment tissue samples allowed us to examine gene expression changes in response to treatment. Recent studies have focused on using DNA microarrays to identify gene expression patterns predictive of chemotherapy response (Chang *et al*, 2003; Ayers *et al*, 2004; Bertucci *et al*, 2004; Hannemann *et al*, 2005; Iwao-Koizumi *et al*, 2005; Sørlie *et al*, 2006), and we performed similar analyses here.

## PATIENTS AND METHODS

### Eligibility criteria

Female patients 18 years or older with a histopathological or cytological diagnosis of breast carcinoma were eligible. Patients with T3, T4, N2, or T2 tumours ⩾3 cm were included. No prior treatments were permitted. Additional inclusion criteria were Karnofsky performance status ⩾70, adequate bone marrow reserve and organ function (hepatic and renal), and normal left ventricular ejection fraction (67±8%) by echocardiography. Patients with inflammatory breast cancer, a second primary malignancy, a previous cancer within the last 5 years, or active cardiac disease not controlled by therapy and/or an infarction within the preceding 6 months were excluded. Appropriate ethical review boards approved the protocol, patients provided written informed consent before enrolment, and the trial was conducted in accordance with the Declaration of Helsinki and good clinical practise standards.

### Study design

The primary objective of this multicentre, open-label, non-randomised, phase II study was to evaluate the pCR rate of sequential neoadjuvant Gem+Dox followed by Gem+Cis in patients with operable breast cancer. The secondary objectives of the study were to characterise toxicity, to examine gene expression profiles and identify biomarkers that might predict and/or characterise chemotherapy response, and to determine survival and progression-free survival rates after 5 years. Because patients remain in follow-up at the time of this publication, time-to-event parameters will be presented in a subsequent publication.

### Treatment plan

Treatment consisted of four cycles (21 days) of gemcitabine 1200 mg m^−2^ plus doxorubicin 60 mg m^−2^ (Gem+Dox), four cycles of gemcitabine 1000 mg m^−2^ plus cisplatin 70 mg m^−2^ (Gem+Cis), and surgery. Gemcitabine, doxorubicin, and cisplatin were administered by intravenous infusion and cisplatin administration included standard hydration and antiemetic procedures. Full supportive care, including antibiotics, antiemetics, and granulocyte colony-stimulation factors, was administered as clinically indicated.

We administered three sequences of Gem+Dox during cycle 1 and collected tumour biopsies after the first dose of treatment via fine needle aspiration cytology to capture single-agent treatment effects and potential changes in molecular profiles. The first 20 patients received gemcitabine on days 1 and 8 plus doxorubicin on day 2 (Gem d1, 8; Dox d2). The next 20 patients received gemcitabine on days 1 and 8 plus doxorubicin on day 1 (Gem d1, 8; Dox d1). The remaining 25 patients received doxorubicin on day 1 plus gemcitabine on days 2 and 8 (Gem d2, 8; Dox d1). For cycles 2 through 4, all patients received Gem d1, 8 and Dox d1. For cycles 5 through 8, patients received gemcitabine on days 1 and 8 plus cisplatin on day 1 (Gem+Cis).

Day-1 doses for gemcitabine, doxorubicin, or cisplatin were delayed for 1 week if the absolute neutrophil count (ANC) was <1.5 × 10^9^ l^−1^ and/or platelets were <100 × 10^9^ l^−1^. Subsequent gemcitabine and cisplatin doses were decreased by 25% after any of the following: febrile neutropenia, grade 4 neutropenia lasting >7 days, grade 4 thrombocytopenia lasting >3 days, ⩾grade 2 bleeding with thrombocytopenia (any grade), or grade 3 non-haematologic toxicities (except nausea/vomiting). Grade 4 non-haematologic toxicities required either a 50% dose reduction of gemcitabine and cisplatin or a delay. Cisplatin doses were reduced by 50% for grade 2 peripheral neurotoxicity or creatinine clearance between 35 and 49 ml min^−1^; the cycle was delayed for grade 3 or 4 peripheral neurotoxicity or creatinine clearance <35 ml min^−1^. A decrease in ejection fraction below 45% or a net decrease in ejection fraction ⩾10% below baseline, or clinical congestive heart failure required patient discontinuation.

Gemcitabine day-8 doses were reduced by 25% if the ANC was between 0.5 and 0.99 × 10^9^ l^−1^ or platelets were between 50 and 99 × 10^9^ l^−1^. Gemcitabine day-8 doses were omitted if the ANC was <0.5 × 10^9^ l^−1^ and platelets were >50 × 10^9^ l^−1^, or for any ANC and platelets <50 × 10^9^ l^−1^. Day-8 doses were reduced by 50% or omitted for grade 3 non-haematologic toxicities (except nausea/vomiting), and were omitted for grade 4 non-haematologic toxicities (except nausea/vomiting). Patients who required either a 6-week dose delay or three dose reductions were discontinued from treatment.

The extent and type of surgery that followed chemotherapy (breast conservation surgery or mastectomy with axillary lymph node dissection) was guided by the tumour size, physician, and/or patient decision. After surgery, patients who were oestrogen receptor- and/or progesterone receptor-positive were treated with tamoxifen.

### Baseline and treatment assessments

Pretreatment biopsies (incisional) were taken for histopathology, prognostic marker evaluation (hormone receptor and HER2), and DNA microarray analysis. Hormone receptors (oestrogen receptor or progesterone receptor) and HER2 status were determined by immunohistochemistry. Two additional samples, one fine needle aspiration cytology collected before treatment on day 2 of cycle 1 and one needle core biopsy collected at the end of cycle 4, were collected for future correlative analyses.

Baseline radiological imaging studies (mammogram, abdominal ultrasound, and chest *x*-ray) were performed. Clinical response, evaluated by physical exam, was classified using the Southwest Oncology Group criteria (Green and Weiss, 1992) before each cycle for all patients who received at least one dose of chemotherapy. Pathologic response was assessed using the Miller and Payne classification system (Ogston *et al*, 2003), with a pCR defined as grade 5. Specifically, a grade 5 designation is characterised as no malignant cells in sections from the site of the previous tumour and allows for the presence of ductal carcinoma *in situ*. The pathologic response status was evaluated by a local pathologist and an independent reviewer. All patients who had surgery after chemotherapy were included in the assessment of pathologic response.

Clinical laboratory tests (haematology and blood chemistries) were performed at baseline, before the day-8 dose of each cycle, within 4 days before the start of subsequent cycles, and on day 21 of cycle 8. All women who received at least one dose of chemotherapy were assessed for safety before each cycle using the National Cancer Institute Common Toxicity Criteria Scale, version 2.0 (National Cancer Institute, 1999).

### Statistical considerations

Potential clinical prognostic factors (age, menopausal status, tumour size, clinical node status, hormone receptor status, and HER2 status) were assessed individually for their association with both clinical response and pCR using logistic regression analysis.

### RNA isolation and microarray hybridisation

Pretreatment incisional biopsies were immediately placed into RNAlater® (Ambion, Foster City, CA, USA) and stored at −80 °C. Total RNA was prepared from biopsies using Qiagen RNAeasy kits (Qiagen, Valencia, CA, USA) and its quality was checked using an Agilent Bioanalyzer (Agilent, Santa Clara, CA, USA). Samples with ⩾1 *μg* of total RNA and discernable 18S and 28S peaks were used for microarray analysis (46 of 65 patients). Total RNA was amplified and labelled as previously described (Hu *et al*, 2005). Microarray hybridisations were performed on Agilent Human 1A (V2) microarrays using 2 *μg* of Cy3-labelled common reference sample (Novoradovskaya *et al*, 2004) and 2 *μg* of Cy5-labelled experimental sample. Microarrays were hybridised overnight, washed, dried, and scanned as previously described (Hu *et al*, 2005). Microarray image files were analysed with GenePix Pro 4.1 (Molecular Devices, Sunnyvale, CA, USA) and loaded into the UNC-CH Microarray Database (https://genome.unc.edu/), and are available in the Gene Expression Omnibus (http://www.ncbi.nlm.nih.gov/geo) under the series numbers GSE8465.

### Microarray analysis and prediction of response

Data from the microarray experiments were processed as previously described (Hu *et al*, 2005). Briefly, genes that did not have a signal intensity ⩾30 in both channels for ⩾70% of the experiments were excluded. To predict response, the gene expression data for the 46 pretreatment samples were analysed using the following as ‘supervising parameters’: clinical complete response (CR) after each cycle of chemotherapy (CR *vs* PR, SD, and PD combined), overall clinical complete response (CR *vs* non-CR, evaluated after the last successfully completed therapy cycle, not necessarily cycle 8), pCR, and clinical ER status (included as a positive control).

Four statistical classification methods were used to predict chemotherapy response using the pretreatment gene expression data: a k-nearest neighbour classifier (k-NN with *k*=1, 3, 5, or 7) with either Euclidean distance or one minus Spearman correlation as the distance function and a Class Nearest Centroid (CNC) classifier with either Euclidean distance or one minus Spearman correlation as the distance function, as described previously (Chung *et al*, 2004). To evaluate prediction accuracy, each of the four classification methods underwent 10-fold cross validation (CV); in each round of CV, each predictor using *n* genes (how the *n* genes were selected is described below) was trained on 90% of the samples and used to make predictions on the remaining 10%, with this procedure repeated nine more times such that every sample was ‘left out’ exactly once. The mean prediction accuracy for the 10 iterations was recorded for each classification method using *n* genes. Note that *n* was increased for subsequent rounds of CV. For each response variable, the set of *n* genes that gave the highest average prediction accuracy during CV was determined and reported for each classification method (accuracies were reported with associated binomial confidence intervals).

Each classification method required a gene/feature selection step to identify genes associated with each ‘class’ (i.e., CR *vs* PR+SD). For all four methods, we used a gene selection method described by Dudoit *et al* (2002); the genes were identified in the training set according to the ratio of between-class to within-class sums of squares. The top *n*-ranked genes were used during each round of CV. Because the number of cases in our study was relatively small (*n*=46), we did not break our data into training and test sets but instead performed 10-fold CV using the four statistical classification methods to avoid overfitting caused by using a single classification method or fortuitous training and test set randomisations.

## RESULTS

### Patient characteristics

Sixty-five Indian women enroled between February 2003 and March 2004 at the All India Institute of Medical Sciences in New Delhi, the Christian Medical College and Hospital in Vellore, and the Hirabai Cowasji Jehangir Medical Research Institute, Jehangir Hospital, in Pune. Table 1 shows baseline patient and disease characteristics. Most patients had ductal breast cancer (61 of 65). Patients generally had large tumours, with 84% of primary tumours identified as T3 or larger, and the median size of the largest baseline lesion was 30 cm^2^ (range, 7.8–85.8 cm^2^).

### Treatment administration

Fifty-three patients had post-treatment surgery, but only 40 of these completed all eight cycles of chemotherapy. Twenty-five patients (38.5%) discontinued before completing eight cycles, although 13 of these had surgery. Twelve of 25 patients who discontinued stopped on or before cycle 4. Reasons for discontinuing chemotherapy included: adverse event (5 of 25), death from study drug toxicity (4 of 25), unrelated death (1 of 25), disease progression (2 of 25), satisfactory response (9 of 25, and all but one of these had surgery), and other patient decision (4 of 25). For the subset of patients who discontinued without surgery (12 of 25), reasons for discontinuation included death (5 of 12), adverse event (2 of 12), and missing follow-up visits or other patient decision (5 of 12).

The median relative dose intensity was 97% for doxorubicin, 96% for gemcitabine in cycles 1 through 4, 86% for gemcitabine in cycles 5 through 8, and 95% for cisplatin. There were 49 dose reductions (37 gemcitabine, seven doxorubicin, and five cisplatin) due to adverse events, with neutropenia (25 of 49) and febrile neutropenia (15 of 49) being the most prevalent reasons for adjustment. There were 34 gemcitabine dose omissions attributed to adverse events, most of which were due to neutropenia (13 of 34), fatigue (6 of 34), or mucositis (3 of 34). Of the 33 cycle delays attributed to adverse events, most were due to neutropenia, leukopenia, or mucositis.

### Clinical results

#### Efficacy

Patient outcomes are summarised in Table 2. The overall clinical response rate was 81.5% (with 22 CRs), and all of the responders showed the first signs of response on or before cycle 4. After completion of therapy, 35 patients had modified radical mastectomy and 18 had breast conservation surgery. Of the 53 patients who had surgery, 13 (24.5%) showed a pCR.

Prognostic factor analyses conducted on all enroled patients showed that age, menopausal status, hormone receptor status, HER2 status, tumour size, and clinical node status were not associated with either clinical response (CR or PR) or pCR.

#### Toxicity

Although subgroup analyses of cohorts that had alternate cycle-1 dosing sequences were not preplanned, unforeseen safety issues prompted a more detailed evaluation. Patients who received Gem d1, d8 and Dox d2 during cycle 1 showed higher rates of toxicity. This was unexpected since the three patient cohorts received the same treatment schedule for Gem+Dox in cycles 2 through 4 (Gem d1, 8; Dox d1) and for Gem+Cis in cycles 5 through 8. Supplementary Table 1 lists the incidence of toxicity by cycle for each cycle-1 cohort. Eighty per cent of the CTC grade 3 or 4 (G3/4) toxicities seen in cycle 1 occurred in the patients who received Gem d1, 8 and Dox d2 (33 of 41). The most common cycle-1 toxicities for this cohort were febrile neutropenia or infection with G3/4 neutropenia in 35% of patients (three patients with G3 and four patients with G4) and G3/4 vomiting in 25% of patients (three patients with G3 and two patients with G4). G3 dehydration (3 of 20), G3 diarrhoea (3 of 20), and G3 thrombocytopenia (3 of 20) each occurred in 15% of patients in cycle 1.

A summary of G3/4 toxicity reported throughout the entire study is shown in Table 3. Neutropenia was prevalent in all three cohorts, with an incidence of 23.1% for grade 3 (15 of 65) and 18.5% (12 of 65) for grade 4. Both G3 mucositis and G3/4 anaemia were somewhat more prevalent in the Gem d1, 8 and Dox d2 cohort, but the incidence for each cohort was no higher at the beginning than at the end of the study.

Five patients died on-study and four deaths were attributed to treatment-related toxicity. These deaths did not appear to be directly related to the alternate dosing schedules used in cycle 1. For one patient, the doses for Gem+Dox were incorrectly calculated in cycle 1 such that the patient received a 20% higher dose for gemcitabine and a 25% higher dose for doxorubicin. The patient was hospitalised for neutropenic sepsis several days after the day-8 dose of gemcitabine and died. The second patient experienced neutropenia with infection, hypoglycemia, and anaemia during cycle 3 and died of cardiac arrest. The third patient died after 8 days of hospitalisation in cycle 8 for convulsions, vomiting, diarrhoea, and neutropenic sepsis. The fourth patient was hospitalised during cycle 8 for severe diarrhoea and died of cardiac arrest secondary to severe hypokalemia. A fifth patient died of acute myocardial infarction, which was not attributed to study treatment.

### Gene expression analysis and prediction of response

#### Analysis of tumour samples using the breast intrinsic gene set

We assayed 46 pretreatment biopsy samples using Agilent human microarrays covering over 17 000 genes (19 of 65 samples failed to give usable RNA). To investigate the expression data, we first hierarchically clustered (Eisen *et al*, 1998) the 46 pretreatment samples using a 1300-gene breast intrinsic gene set developed by Hu *et al* (2006), which can identify the intrinsic tumour subtypes (luminal A, luminal B, basal-like, HER2+/ER− and normal breast-like) that show significant differences in patient outcome (Sørlie *et al*, 2001, 2003). The main intrinsic subtypes of luminal, basal-like, and HER2+/ER− (Supplementary Figure 1) were identifiable in this Indian patient dataset, which is consistent with previous studies showing that the breast tumour intrinsic subtypes are conserved across ethnic groups (Yu *et al*, 2004; Carey *et al*, 2006).

#### Association of response with breast intrinsic subtype

We examined response rates within the breast intrinsic subtypes as assigned from Supplementary Figure 1. The basal-like subtype had the highest pCR rate (7 of 14, 50%), which is consistent with findings of previous studies (Rouzier *et al*, 2005; Carey *et al*, 2007), and luminal tumours had the lowest pCR rate (3 of 15, 20%), but the association was not statistically significant (Table 4, *P*=0.23).

#### Prediction of neoadjuvant chemotherapy response

We performed supervised analyses on the pretreatment gene expression data and determined the 10-fold CV error rates for predicting (1) pCR, (2) overall clinical complete response (CR *vs* non-CR), and (3) CR *vs* non-CR after each cycle (results presented here are only for CR *vs* non-CR after cycle 8). Supplementary Tables 2 through 4 show that the 10-fold CV analyses using the CNC and k-NN classification methods yielded gene expression profiles/predictors that accurately classified tumours according to (1) pCR *vs* no pCR (73% (95% CI: 0.57–0.85) −78% [95% CI: 0.63–0.89] accuracy), (2) overall CR *vs* non-CR (83% [95% CI: 0.69–0.92] −88% [95% CI: 0.75–0.95] accuracy), and (3) CR *vs* non-CR after cycle 8 (75% [95% CI: 0.57–0.87] −89% [95% CI: 0.73–0.96] accuracy) for the 46 tumour samples that yielded pretreatment microarray data. Each of the four prediction methods achieved similar accuracies when used to predict a given response variable. Prediction of ER status was included as a positive control for our gene expression-based predictors; it represents the upper threshold of how good a predictor can be (87% [95% CI: 0.74–0.94] −89% [95% CI: 0.76–0.95] accuracy) on this dataset, providing a benchmark against which the chemotherapy response predictors can be judged (Supplementary Table 5).

**Figure 1:**
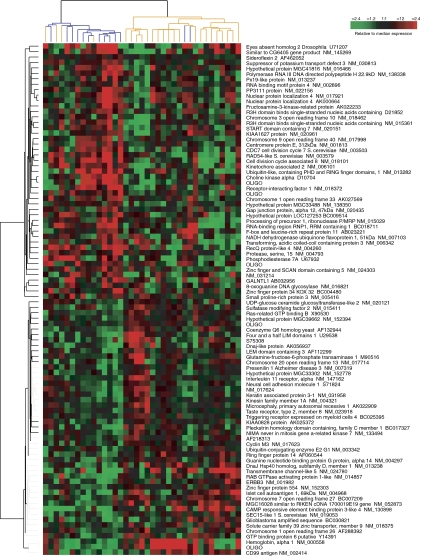
Hierarchical cluster analysis of pretreatment tumour samples using the 94-gene set predictive of pCR. Blue and yellow dendrogram branches indicate tumours with pCR and no pCR, respectively.

**Figure 2:**
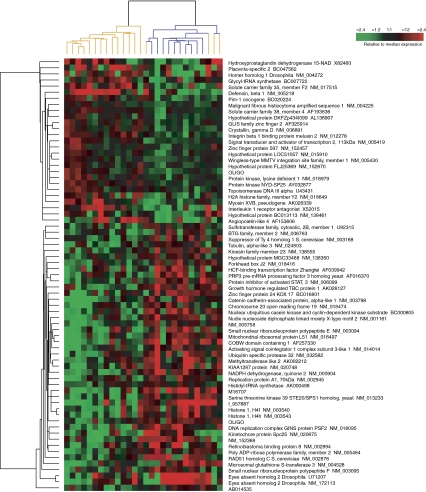
Hierarchical cluster analysis of pretreatment tumour samples using the 71-gene set predictive of clinical response after eight treatment cycles. Blue and yellow dendrogram branches indicate complete and non-complete responders, respectively.

Using the gene lists identified in the 10-fold CV analysis as being predictive of pCR (Supplementary Table 2, Euclidian Nearest Centroid), we hierarchically clustered (Eisen *et al*, 1998) the pretreatment biopsy samples to better understand the predictive genes and their relationships to each other. Figure 1 shows the hierarchical clustering of tumours using the 94-gene set predictive of pCR (76% accuracy in the 10-fold CV analysis). (Note: the classification of samples into clusters and the associated accuracies shown in Figures 1 and 2 and Supplementary Figure 2 are different from those observed in the 10-fold CV analysis and are for illustrative purposes only.) Using the program EASE (Hosack *et al*, 2003), the Gene Ontology (GO) (http://www.geneontology.org) categories ‘nucleotide/nucleic acid metabolism’ and ‘DNA metabolism’ were over-represented relative to chance in the gene set highly expressed in tumours with pCR (Figure 1, top gene dendrogram branch).

Figure 2 shows the hierarchical clustering of tumours using the 71-gene set predictive of clinical response at treatment cycle 8 (86% accuracy in the 10-fold CV analysis). According to EASE, the GO categories ‘nucleotide/nucleic acid metabolism,’ ‘RNA metabolism,’ ‘DNA repair,’ and ‘response to stress’ were over-represented relative to chance in the gene set highly expressed in tumours with CR at cycle 8 (Figure 2, bottom gene dendrogram branch). Interestingly, the complete responders at cycle 8 showed high expression of NUDT2, which is not only involved in nucleotide metabolism but may also promote apoptosis (Vartanian *et al*, 1999; Swarbrick *et al*, 2005). Supplementary Figure 2 shows the hierarchical clustering of tumours using the 66-gene set predictive of overall CR (88% accuracy in the 10-fold CV analysis). Interestingly, the responders represented in Supplementary Figure 2 showed high expression of FADD, which also promotes apoptosis (Chinnaiyan *et al*, 1995).

## DISCUSSION

This phase II study of sequential Gem+Dox and Gem+Cis as neoadjuvant therapy in breast cancer showed strong activity (an overall clinical response rate of 81.5% and a pCR rate of 24.5%), but also revealed unforeseen safety issues related to the administration of an alternate schedule of Gem+Dox in cycle 1. To facilitate tissue sampling for biomarker analyses in the present trial, we used three dosing sequences in cycle 1 only. Dosing sequences were Gem d1, d8 and Dox d2 or Gem d2, d8 and Dox d1 in two of the three patient cohorts, and the more standard dosing sequence of Gem d1, d8 and Dox d1 for the third cohort in cycle 1 and for all patients in cycles 2 through 4. Patients who received Gem d1, d8 and Dox d2 during cycle 1 showed higher rates of toxicity during cycle 1 (Supplementary Table 1), with febrile neutropenia, neutropenia with infection, and vomiting being the most prevalent toxicities. Toxicity profiles for the other two cohorts were more manageable (Table 3), and the incidence and types of adverse events were similar to those in previous phase II studies of gemcitabine plus doxorubicin in patients with breast cancer (Pérez-Manga *et al*, 2000; Gomez *et al*, 2001; El Serafy *et al*, 2003; Bensalem and Bouzid, 2006).

Although several different dosing regimens of Gem+Dox were applied in previous studies, they were all similar in that gemcitabine and doxorubicin were both given on day 1 and, in some cases, were given together again on day 8 (Pérez-Manga *et al*, 2000; Gomez *et al*, 2001; El Serafy *et al*, 2003; Bensalem and Bouzid, 2006). In these previous studies, haematologic toxicities were the most common, but alopecia and mucositis were also prevalent. We know of no other studies that have evaluated the administration of gemcitabine and doxorubicin sequentially on days 1 and 2, and these results suggest that this schedule induces unacceptable toxicity. The number of treatment-related deaths was also high in this study, but the affected patients were not disproportionately associated with any single cohort. The details of each case were evaluated thoroughly, and we were unable to identify unifying characteristics that indicated why these individuals were more vulnerable to the serious side effects of chemotherapy. Notably, while the standard Gem+Dox dose schedule produced a toxicity profile that was similar to previous studies with Gem+Dox, the toxic death rate was higher than that in previous trials (Pérez-Manga *et al*, 2000; Gomez *et al*, 2001; El Serafy *et al*, 2003; Bensalem and Bouzid, 2006). These events highlight the need for appropriate patient education, careful screening, and the use of additional supportive services in developing countries like India, especially in areas where health-care access may be limited.

The identification of molecular predictors of patient outcomes will help us design safer and more effective regimens that are tailored to individual patients. In the present study, we determined that pretreatment gene expression patterns could predict response to gemcitabine-containing neoadjuvant therapy. The accuracy rates and associated confidence intervals achieved were encouraging and similar to those achieved by predictors developed for other neoadjuvant regimens (Chang *et al*, 2003; Ayers *et al*, 2004). pCR is strongly correlated with improved long-term disease-free and overall survival (Fisher *et al*, 1998; Kuerer *et al*, 1999; Wolmark *et al*, 2001). There is evidence that clinical complete response at the end of neoadjuvant chemotherapy is also correlated with improved long-term survival (Ferrière *et al*, 1998; Chang *et al*, 1999; Pierga *et al*, 2003; Cleator *et al*, 2005). Thus, the gene expression-based predictors obtained in this study for pCR, overall clinical complete response, and clinical complete response after cycle 8 have the potential to be clinically useful if further validated. As might be expected from gemcitabine's mechanism of action, nucleotide metabolism signatures were found to be associated with response.

To conclude, sequential neoadjuvant gemcitabine plus doxorubicin followed by gemcitabine plus cisplatin was effective for downstaging large breast tumours in patients with operable breast cancer, thereby improving treatment options for patients who desired breast conservation surgery instead of mastectomy. Future results for overall and disease-free survival will determine if these high response rates will translate into improved long-term efficacy. Significant toxicity was observed in a subgroup of patients who received Gem d1, d8 and Dox d2, and this dosing sequence is not recommended. The gene expression-based predictors identified here may provide a method for selecting patients most likely to benefit from gemcitabine-containing neoadjuvant therapy and, thus, warrant further validation using additional datasets as they emerge.

## Figures and Tables

**Table 1:** Baseline patient and disease characteristics

	**Gem d1,8; Dox d2^a^(*n*=20)**	**Gem d1,8; Dox d1^a^(*n*=20)**	**Gem d2,8; Dox d1^a^(*n*=25)**	**All(n=65)^b^**	**Correlative Subset(*n*=46)^c^**
Median age (range), years	45.5 (35–65)	45.5 (31–69)	49 (31–70)	46 (31–70)	48 (32–69)
*Karnofsky performance status, n (%)*					
90	19 (95.0)	17 (85.0)	20 (80.0)	56 (86.2)	40 (87.0)
100	1 (5.0)	3 (15.0)	5 (20.0)	9 (13.8)	6 (13.0)
*Menstruation status, n (%)*					
Premenopausal	8 (40.0)	10 (50.0)	11 (44.0)	29 (44.6)	19 (41.3)
Postmenopausal	12 (60.0)	10 (50.0)	14 (56.0)	36 (55.4)	27 (58.7)
*Disease stage, n (%)*					
IIA	2 (10.0)	0	1 (4.0)	3 (4.6)	2 (4.3)
IIB	10 (50.0)	9 (45.0)	11 (44.0)	30 (46.2)	21 (45.7)
IIIA	4 (20.0)	6 (30.0)	8 (32.0)	18 (27.7)	11 (23.9)
IIIB	4 (20.0)	5 (25.0)	5 (20.0)	14 (21.5)	12 (26.1)
					
*Estrogen receptor/progesterone receptor status, n (%)*					
+/+	4 (20.0)	10 (50.0)	13 (52.0)	27 (41.5)	19 (41.3)
+/−	4 (20.0)	3 (15.0)	1 (4.0)	8 (12.3)	5 (10.9)
−/+	1 (5.0)	0	2 (8.0)	3 (4.6)	3 (6.5)
−/−	11 (55.0)	6 (30.0)	8 (32.0)	25 (38.5)	18 (39.1)
					
*HER2-neu expression, n (%)*					
0, or 1+ or 2+	7 (35.0)	13 (65.0)	17 (68.0)	37 (56.9)	27 (58.7)
3+	9 (45.0)	5 (25.0)	5 (20.0)	19 (29.2)	12 (26.1)
Not detected	4 (20.0)	1 (5.0)	2 (8.0)	7 (10.8)	6 (13.0)

d=day; Dox=doxorubicin; Gem=gemcitabine; y=years.

a Cohort defined by cycle-1 schedule of gemcitabine plus doxorubicin.

b Total N equals 63 for hormone receptor status and HER2-neu expression due to insufficient sample quantity. Note that 12 of 63 patients in the trial and 10 of 45 in the correlative subset were negative for receptor status and HER2 (i.e., HER2 was either not detected or 0).

c Correlative subset includes patients with pretreatment microarray data. Correlative subset equals 45 for receptor status and HER2-neu expression.

**Table 2:** Patient outcomes after neoadjuvant chemotherapy

	**Correlative subset^a^(*n*=46)**	**All(n=65)**
Overall clinical response (CR+PR)^b^, *n* (%)	37 (80.4)	53 (81.5)
CR	16 (34.8)	22 (33.8)
PR	21 (45.7)	31 (47.7)
SD	5 (10.9)	7 (10.8)
Not determined	4 (8.7)	5 (7.7)
Surgery, *n* (%)	37 (80.4)	53 (81.5)
Pathologic complete response (pCR)^c^, *n* (%)	13 (35.1)	13 (24.5)

CR=complete response; PR=partial response; SD=stable disease.

a Correlative subset includes patients with pretreatment microarray data. Twenty-eight of these patients completed all eight cycles of chemotherapy and had surgery.

b SWOG overall best study response rates were based on all enroled patients who received at least one dose of chemotherapy. SWOG best response was not determined for patients who discontinued treatment after one or two cycles.

c The pCR rate was based on the total number of patients who had posttreatment pathology (i.e., 37 for the correlative subset and 53 for all patients).

**Table 3:** Incidence of CTC grade 3 and grade 4 haematologic and non-haematologic toxicity by cohort^a^

	**Gem d1,8; Dox d2 (*n*=20)**		**Gem d1,8; Dox d1 (*n*=20)**		**Gem d2,8; Dox d1 (*n*=25)**	
**Toxicity^b^**	**Grade 3**	**Grade 4**	**Grade 3**	**Grade 4**	**Grade 3**	**Grade 4**
Anaemia	5	3	1	1	1	1
Neutropenia	5	7	6	3	4	2
Leukopenia	2	0	1	0	0	0
Thrombocytopenia	4	1	2	0	0	1
ALT (SGPT)	1	0	0	0	0	1
Hypokalemia	1	3	0	1	0	0
Hyponatremia	1	0	0	1	1	0
Hypernatremia	0	1	0	0	0	0
Hypoglycemia	0	0	0	0	0	1
Fatigue	2	0	1	1	1	0
Oral/pharyngeal mucositis	5	1	0	0	2	0
Dehydration	5	0	0	0	1	0
Vomiting	3	3	0	0	1	0
Diarrhoea	4	0	1	0	0	0
Febrile neutropenia	3	4	0	0	0	0
Infection with grade 3/4 neutropenia	1	1	0	2	0	1

ALT=alanine aminotransferase; d=day; Dox=doxorubicin; Gem=gemcitabine; SGPT=serum glutamic pyruvic transaminase.

a Cohort defined by cycle-1 schedule of gemcitabine plus doxorubicin.

b Toxicities were graded according to Common Toxicity Criteria (version 2.0) and listed according to maximum grade reported. Includes all toxicities recorded as grade 4 and all grade 3 toxicities that occurred in >2 patients.

**Table 4:** Association between response designation and tumour intrinsic subtype for the correlative subset (*n*=46)^a^

	**Pathologic Complete Response^b^**		**Clinical Response Designation^b^**		
**Intrinsic subtype**	**Yes**	**No**	**CR**	**PR**	**SD**
Luminal (A+B)	3	12	7	8	1
HER2+/ER−	3	4	1	6	1
Basal-like	7	7	7	7	3

a Correlative subset includes patients with pretreatment microarray data.

b Clinical response was not determined for four patients who discontinued after one or two cycles. One patient with a CR was not included in the table because classification was normal breast-like.
