# Supplementary material for: A phase II study of sequential neoadjuvant gemcitabine plus doxorubicin followed by gemcitabine plus cisplatin in patients with operable breast cancer: prediction of response using molecular profiling
Source: Br J Cancer. 2008 Apr 1;98(8):1327–35. doi: 10.1038/sj.bjc.6604322 (PMC2361717; doi:10.1038/sj.bjc.6604322)
Supplement: Supplementary Tables 1–5 and Figures Legend [file 6604322x2.doc]

# Supplementary information

Supplementary Table 1. Incidence of CTC Grade 3/4 Toxicity by Cycle and Patient Cohort^a^

|  | **Number of Grade 3/4 Events^b^** | | | |
| --- | --- | --- | --- | --- |
| **Cycle** | **Gem d1,8; Dox d2** | **Gem d1,8; Dox d1** | **Gem d2,8; Dox d1** | **Total** |
| 1^c^ | 33 | 5 | 3 | 41 |
| 2^d^ | 2 | 0 | 1 | 3 |
| 3^d^ | 4 | 1 | 5 | 10 |
| 4^d^ | 7 | 2 | 2 | 11 |
| 5^e^ | 3 | 2 | 3 | 8 |
| 6^e^ | 1 | 3 | 1 | 5 |
| 7^e^ | 10 | 6 | 3 | 19 |
| 8^e^ | 16 | 6 | 4 | 26 |
| Total | 76 | 25 | 22 | 123 |

^a^Cohort defined by cycle-1 schedule of gemcitabine plus doxorubicin.

^b^Reported as the first occurrence of the maximum severity grade experienced per patient.

^c^For cycle 1, patients received Gem d1,8; Dox d2 (n=20), Gem d1,8; Dox d1 (n=20), and Gem d2,8; Dox d1 (n=25).

^d^For cycles 2-4, all patients received Gem d1,8; Dox d1.

^e^For cycles 5-8, all patients received gemcitabine d1,8; cisplatin d1.

Supplementary Table 2. Prediction of pCR (13 pCRs vs 24 non-pCRs)

| **Classification Method** | **Gene #** | **Accuracy (95% CI)** | **True +^a^** | **True –^b^** | **False +^c^** | **False –^d^** | **Sens.** | **Spec.** | **PPV** | **NPV** |
| --- | --- | --- | --- | --- | --- | --- | --- | --- | --- | --- |
| Spearman Nearest Centroid | 41 | 0.730 (0.570, 0.846) | 12 | 15 | 9 | 1 | 0.923 | 0.625 | 0.571 | 0.938 |
| Euclidean Nearest Centroid^e^ | 94 | 0.757 (0.599, 0.866) | 10 | 18 | 6 | 3 | 0.769 | 0.750 | 0.625 | 0.857 |
| Spearman  k-NN (k=3) | 99 | 0.784 (0.628, 0.886) | 11 | 18 | 6 | 2 | 0.846 | 0.750 | 0.647 | 0.900 |
| Euclidean k-NN (k=3) | 93 | 0.784 (0.628, 0.886) | 10 | 19 | 5 | 3 | 0.769 | 0.792 | 0.667 | 0.864 |
| Average |  | 0.764 (0.606, 0.871) |  |  |  |  | 0.827 | 0.729 | 0.628 | 0.890 |

pCR=pathologic complete response; CI=confidence interval; sens.=sensitivity; spec.=specificity; PPV=positive predictive value; NPV=negative predictive value; k-NN=k-nearest neighbors.

^a^True + refers to number of responders correctly identified.

^b^True – refers to number of non-responders correctly identified.

^c^False + refers to number of non-responders incorrectly identified as responders.

^d^False – refers to number of responders incorrectly identified as non-responders.

^e^Gene list obtained from the Euclidean Nearest Centroid method was used to cluster tumors in Figure 1.

Supplementary Table 3. Prediction of Overall Complete Response (16 CRs vs 26 non-CRs [21 PRs and 5 SDs])

| **Classification Method** | **Gene #** | **Accuracy (95% CI)** | **True +^a^** | **True –^b^** | **False +^c^** | **False –^d^** | **Sens.** | **Spec.** | **PPV** | **NPV** |
| --- | --- | --- | --- | --- | --- | --- | --- | --- | --- | --- |
| Spearman Nearest Centroid | 29 | 0.833 (0.694, 0.917) | 16 | 19 | 7 | 0 | 1.000 | 0.731 | 0.696 | 1.000 |
| Euclidean Nearest Centroid^e^ | 66 | 0.881 (0.750, 0.948) | 14 | 23 | 3 | 2 | 0.875 | 0.885 | 0.824 | 0.920 |
| Spearman k-NN (k=5) | 29 | 0.833 (0.694, 0.917) | 12 | 23 | 3 | 4 | 0.750 | 0.885 | 0.800 | 0.852 |
| Euclidean k-NN (k=5) | 55 | 0.857 (0.722, 0.933) | 14 | 22 | 4 | 2 | 0.875 | 0.846 | 0.778 | 0.917 |
| Average |  | 0.851 (0.715, 0.929) |  |  |  |  | 0.875 | 0.837 | 0.774 | 0.922 |

CR=clinical complete response; PR=partial response; SD=stable disease; CI=confidence interval; sens.=sensitivity; spec.=specificity; PPV=positive predictive value; NPV=negative predictive value; k-NN=k-nearest neighbors.

^a^True + refers to number of responders correctly identified.

^b^True – refers to number of non-responders correctly identified.

^c^False + refers to number of non-responders incorrectly identified as responders.

^d^False – refers to number of responders incorrectly identified as non-responders.

^e^Gene list obtained from the Euclidean Nearest Centroid method was used to cluster tumors in Figure 2.

Supplementary Table 4. Prediction of Complete Response at Cycle 8 (10 CRs vs 18 non-CRs [17 PRs and 1 SD])

| **Classification Method** | **Gene #** | **Accuracy (95% CI)** | **True +^a^** | **True –^b^** | **False +^c^** | **False –^d^** | **Sens.** | **Spec.** | **PPV** | **NPV** |
| --- | --- | --- | --- | --- | --- | --- | --- | --- | --- | --- |
| Spearman Nearest Centroid | 24 | 0.750 (0.566, 0.873) | 10 | 11 | 7 | 0 | 1.000 | 0.611 | 0.588 | 1.000 |
| Euclidean Nearest Centroid^e^ | 71 | 0.857 (0.685, 0.943) | 8 | 16 | 2 | 2 | 0.800 | 0.889 | 0.800 | 0.889 |
| Spearman k-NN (k=7) | 23 | 0.821 (0.644, 0.921) | 10 | 13 | 5 | 0 | 1.000 | 0.722 | 0.667 | 1.000 |
| Euclidean k-NN (k=7) | 44 | 0.893 (0.728, 0.963) | 9 | 16 | 2 | 1 | 0.900 | 0.889 | 0.818 | 0.941 |
| Average |  | 0.830 (0.656, 0.925) |  |  |  |  | 0.925 | 0.778 | 0.718 | 0.958 |

CR=clinical complete response; PR=partial response; SD=stable disease; CI=confidence interval; sens.=sensitivity; spec.=specificity; PPV=positive predictive value; NPV=negative predictive value; k-NN=k-nearest neighbors.

^a^True + refers to number of responders correctly identified.

^b^True – refers to number of non-responders correctly identified.

^c^False + refers to number of non-responders incorrectly identified as responders.

^d^False – refers to number of responders incorrectly identified as non-responders.

^e^Gene list obtained from the Euclidean Nearest Centroid method was used to cluster tumors in Figure A2.

Supplementary Table 5. Prediction of ER status (24 ER+ vs 21 ER-)

| **Classification Method** | **Gene #** | **Accuracy (95% CI)** | **True +^a^** | **True –^b^** | **False +^c^** | **False –^d^** | **Sens.** | **Spec.** | **PPV** | **NPV** |
| --- | --- | --- | --- | --- | --- | --- | --- | --- | --- | --- |
| Spearman Nearest Centroid | 100 | 0.889 (0.765, 0.952) | 20 | 20 | 1 | 4 | 0.833 | 0.952 | 0.952 | 0.833 |
| Euclidean Nearest Centroid | 100 | 0.889 (0.765, 0.952) | 20 | 20 | 1 | 4 | 0.833 | 0.952 | 0.952 | 0.833 |
| Spearman k-NN (k=3) | 100 | 0.867 (0.738, 0.938) | 19 | 20 | 1 | 5 | 0.792 | 0.952 | 0.950 | 0.800 |
| Euclidean k-NN (k=3) | 100 | 0.889 (0.765, 0.952) | 20 | 20 | 1 | 4 | 0.833 | 0.952 | 0.952 | 0.833 |
| Average |  | 0.884 (0.758, 0.949) |  |  |  |  | 0.823 | 0.952 | 0.952 | 0.825 |

ER=estrogen receptor; CI=confidence interval; sens.=sensitivity; spec.=specificity; k-NN=k-nearest neighbors; PPV=positive predictive value; NPV=negative predictive value.

^a^True + refers to number of responders correctly identified.

^b^True – refers to number of non-responders correctly identified.

^c^False + refers to number of non-responders incorrectly identified as responders.

^d^False – refers to number of responders incorrectly identified as non-responders.

**Supplementary Figure Legends**

**Supplementary Figure 1.** Hierarchical cluster of the 46 tumor samples and five normal breast samples analyzed using the 1300-gene intrinsic breast gene set developed by Hu et al (Hu *et al*, 2006). A scaled-down representation of the complete cluster diagram is shown on the left, and various gene clusters are indicated and shown on the right.

**Supplementary Figure 2.** Hierarchical cluster analysis of pretreatment tumor samples using the 66-gene set predictive of overall clinical response. Blue and yellow dendrogram branches indicate clinical complete and non-complete overall responders, respectively.
