# Supplementary material for: A phase II study of sequential neoadjuvant gemcitabine plus doxorubicin followed by gemcitabine plus cisplatin in patients with operable breast cancer: prediction of response using molecular profiling
Source: Br J Cancer. 2008 Apr 1;98(8):1327–35. doi: 10.1038/sj.bjc.6604322 (PMC2361717; doi:10.1038/sj.bjc.6604322)

Supplemental Figure 2

Julka et al.

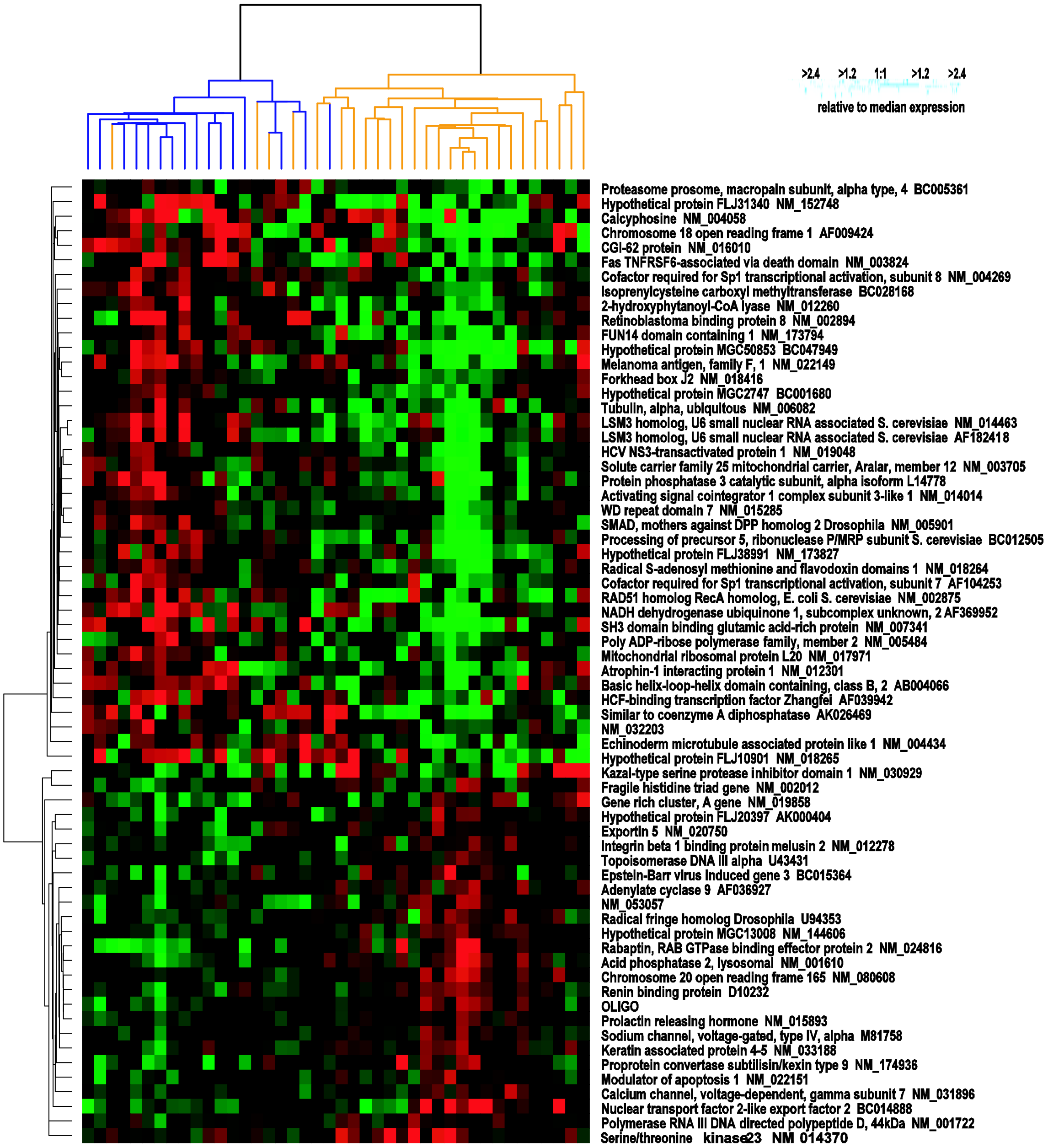

Supplemental Figure 1

Julka et al.

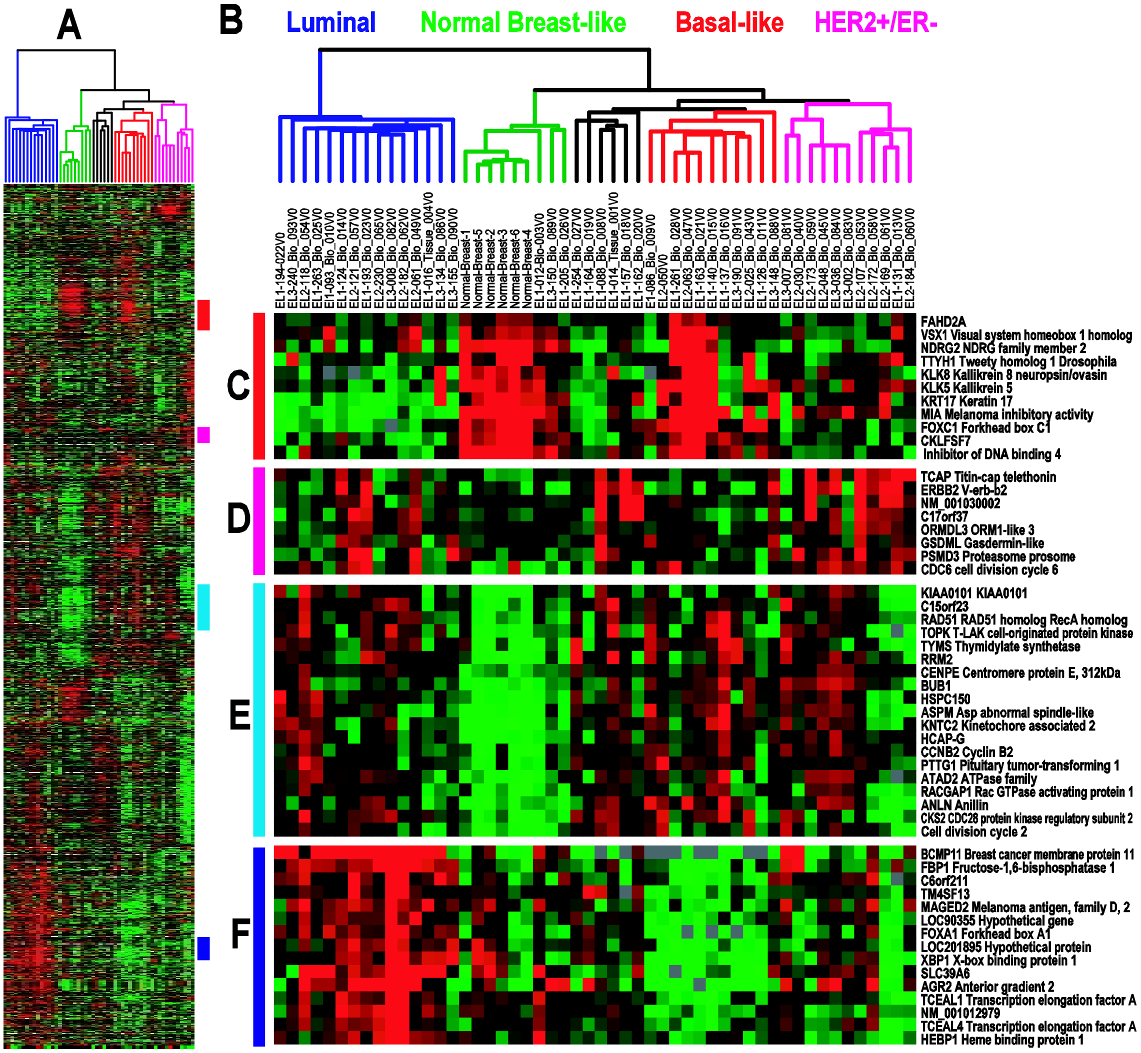

Supplement: Supplementary file 2 [file 6604322x1.doc]
